# Mechanical and thermal properties of mud dauber nests under atmospheric drying

**DOI:** 10.1038/s41598-023-39796-x

**Published:** 2023-08-03

**Authors:** Joon S. Park, Hai Lin, Hussein Alqrinawi

**Affiliations:** Department of Civil and Environmental Engineering, Louisiana State University, Baton Rouge, LA 70803 USA

**Keywords:** Civil engineering, Entomology

## Abstract

Mud dauber wasps construct soil nests to protect their offspring from predators, extreme temperatures, and rainwater. The mechanical and thermal properties of these nests are important for the reproductive success of mud daubers. The previous study showed that the high densities and strengths of mud dauber nests were due to the repetitive tapping and atmospheric drying used by mud daubers during nest construction. This study investigated the effect of atmospheric drying on the mechanical and thermal properties of mud dauber nests. The soil shrinkage curve, elastic modulus, suction stress characteristic curve, soil water retention curve, shear strength, and thermal conductivity function of mud dauber nest soils were measured by performing drying cake tests, direct shear tests, unconfined compression tests, and thermal conductivity measurements. This study showed atmospheric drying could increase Young’s moduli (from hundreds to thousands of kPa), the magnitudes of suction stress (up to 64 kPa), and shear strengths (e.g., unconfined compressive strength increased up to 2100 kPa) of mud dauber nests. The thermal conductivity was reduced by 47% due to atmospheric drying. Investigation of mud dauber nests under atmospheric drying could provide insights and inspiration to improve human manufacturing and manipulation of soils for earthen building construction.

## Introduction

Black and yellow mud daubers build nests using local soil for their offspring^1^. Mud dauber nests have irregular shapes and are composed of several tubular brood cells, where female mud daubers lay eggs^2^. While the eggs transform into adult mud dauber wasps in the nests, which takes several months to a year, mud dauber nests protect the eggs from predators (e.g., parasitoid wasps, bee flies, and birds) and varying atmospheric conditions (e.g., extreme temperature, humidity, and rainwater)^3,4^. Thus, the mechanical and thermal properties of mud dauber nests are crucial to the reproductive success of mud daubers^1,3^.

**Figure 1:**
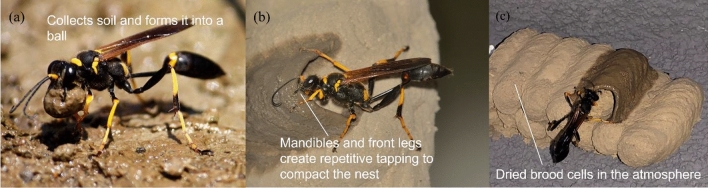
(**a**) A mud dauber forms wet soil into a soil ball for nest construction, (**b**) compacts the nest cell wall by producing a repetitive tapping, and (**c**) starts building a new cell after drying the constructed cells in the atmosphere. (a): photo courtesy of an author named Hglu1 via https://en.wikipedia.org/wiki/Sceliphron_caementarium#/media/File:20100710.MudDauber-SceliphronCaementarium.Hannibal.jpg. The photo is licensed under a Creative Commons Attribution-ShareAlike 3.0 Unported license. The license terms can be found on the following link: https://creativecommons.org/licenses/by-sa/3.0/deed.en. (b): photo used with permission of Peter May. (c): photo courtesy of Dániel Koska via https://www.inaturalist.org/observations/84871363. The photo is licensed under a Creative Commons-NonCommerical 4.0 International license. The license terms can be found on the following link: https://creativecommons.org/licenses/by-nc/4.0/.

The mechanical and thermal properties of mud dauber nests are controlled by the nest construction techniques used by mud daubers^5,6^. Mud dauber can collect sandy silt with some clay for nest building and form it into a soil ball by modulating moisture content (Fig. 1a). During nest construction, mud daubers compact nest cell walls using repetitive tapping produced by the front legs and mandibles (Fig. 1b)^6,7^, which is similar to the vibratory compaction technique used in geotechnical engineering. Once the nest cell is completed, a period of inactivity (about 1 h to 2 days) is purposely given by mud dauber to allow the cell to dry (i.e., atmospheric drying) and increase the nest strength (Fig. 1c)^8^.

The previous study^6^ by the research team reported that the dry densities and penetration resistances of mud dauber nests were similar to the Proctor compacted reconstitute nest soil samples, which is mainly due to the repetitive tapping and atmospheric drying used by mud daubers during nest construction to compact soil and increase soil strength. It is also important to note that mud daubers did not use salivary secretions to cement soil particles during nest construction^6,9^. The similar dry densities and penetration resistances of mud dauber nests to the Proctor compacted nest soil samples demonstrated the high efficiency of mud dauber nest construction techniques. Investigation of mud dauber nests and their construction process could provide insights and inspiration to improve human manufacturing and manipulation of soils (e.g., earthen building construction).

In addition to mud daubers, many animals employ local soils for nest construction (e.g., termites, swallows, horneros, ants, etc.). Their nest constructions often involve atmospheric drying, similar to mud daubers^10–12^. For example, termites and swallows preferably collect low-plasticity soils in a plastic state^10,11^ and assemble them into ongoing nests^10,11^. The nests subsequently undergo strengthening under atmospheric drying condition^13^. As moisture content governs the mechanical and thermal properties of soils, the atmospheric drying process can significantly contribute to the stability and thermal regulations of soil nests and thus the reproductive success of the animals. However, the effect of atmospheric drying on the mechanical and thermal properties of soil nests was not systematically investigated. Given the growing interests in soil nest construction among architects, behavioral ecologists, and entomologists, a comprehensive investigation of this phenomenon can offer valuable insights to researchers who may not be well-versed in soil mechanics. By exploring the influence of atmospheric drying on the mechanical and thermal characteristics of soil nests, this study has the potential to contribute new knowledge to the field of nest construction and foster interdisciplinary collaborations.

The goal of the study is to investigate the effect of atmospheric drying on the mechanical and thermal properties of mud dauber nests using unsaturated soil mechanics principles. The drying cake (DC) method^14^ was used in this study since soil specimens in the DC test are subjected to drying without external confining stresses, which is similar to the mud dauber nests under atmospheric drying. Soil shrinkage curves (SSC) and elastic moduli of nest soils were measured by DC tests to evaluate the volume change and variation of stiffness of mud dauber nests during atmospheric drying. The measured SSC and elastic moduli were then used to calculate the suction stress characteristic curves (SSCC) and the soil water retention curves (SWRC), which were used to describe the internal stress state of nests during atmospheric drying. Direct shear tests and unconfined compression tests were performed to investigate the shear strengths of nest soils at varying moisture contents at the soil sample scale. In addition, the thermal conductivities of nest soils under drying were measured to evaluate the thermal properties of mud dauber nests under atmospheric drying. Lastly, the potential bio-inspiration of mud dauber construction techniques on earthen building construction was discussed.

## Background

### Suction stress characteristic Curve (SSCC), soil water retention curve (SWRC), and thermal conductivity function (TCF)

The mechanical properties of mud dauber nests under atmospheric drying are controlled by the internal stress state, which can be quantified by the suction stress concept proposed by Lu and Likos^15^. Suction stress conceptually represents the stresses acting between soil particles, including capillary force controlled by air–water interfacial surface tension, the physicochemical forces governed by intermolecular interactions between water and soil minerals, and cementation forces (e.g., cement additives)^16^. Following the conceptualization of suction stress, effective stress for unsaturated and saturated soils is given as follows:

1 $${\sigma }{\prime}=\left(\sigma -{u}_{a}\right)-{\sigma }^{s}$$

where $\sigma$ is total stress, ${u}_{a}$ is pore air pressure, and ${\sigma }^{s}$ is suction stress^17^. Suction stress is a negative scalar quantity, indicating suction stress acts as internal tensile stress on the soil skeleton (i.e., attractive stress between soil particles). Therefore, the effective stress equation (Eq. 1) represents the contribution of suction stress to the mechanical properties of unsaturated soils, including volume change, stiffness, and strength^18–26^. Suction stress is dependent on moisture content due to the dependence of physicochemical and capillary forces on moisture content, which is described by the suction stress characteristic curve (SSCC). Numerous studies showed that SSCC governs the mechanical properties of unsaturated soils, such as soil deformation under drying and wetting, shear strength, elastic and shear modulus, and tensile strength^18–21,23–27^. The drying cake (DC) method^14^ was used in this study to measure the SSCC of mud dauber nests.

SSCC is intrinsically related to soil water retention curve (SWRC), which is written by

2 $${\sigma }^{s}={-S}_{e}({u}_{a}-{u}_{w})$$

where ${u}_{a}-{u}_{w}$ is matric suction, ${u}_{w}$ is pore water pressure, and ${S}_{e}$ is effective degree of saturation^17^. SWRC is generally characterized into four regimes, capillary, funicular, pendular, and hydration by the formation of water phase at the pores^16,28^. The SWRC and its regimes can be distinguished by the fitting parameters of the van Genuchten model (1980)^29^, which can be written by

3 $${S}_{e}=\frac{S-{S}_{r}}{1-{S}_{r}}={\left\{\frac{1}{1+{\left[\alpha \left({u}_{a}-{u}_{w}\right)\right]}^{n}}\right\}}^{1-1/n}$$

where $\alpha$ and $n$ are fitting parameters. $\alpha$ is the inverse of the air entry suction (*S*_*AEV*_) at which the air–water interface for surface tension begins to appear in the pores. $n$ is the pore size distribution parameter representing the breadth of pore size distribution. ${S}_{r}$ is the residual degree of saturation (i.e., adsorption capacity) below which the pore water exists as thin films surrounding the soil particle surface due to adsorption^30^. The transition from the capillary to the funicular regime is usually captured at the degree of saturation corresponding to *S*_*AEV*_. The transition from the funicular to the pendular regime is controlled by the pore size distribution parameter *n*. *S*_*r*_ is the degree of saturation at which the soil water retention regime is transitioned from the pendular regime to the hydration regime^15^.

Thermal conductivity of soil varies with degree of saturation due to the significant differences of thermal conductivity between three soil phases (e.g., *k*_*mineral*_ ≈ 3 W/m·K > *k*_*water*_ = 0.56 W/m·K at 0 °C > *k*_*air*_ = 0.026 W/m·K), which can be described by thermal conductivity function (TCF). Since the formations of three soil phases at particle contacts controls the heat transfer in soil, TCF has an intrinsic relationship with SWRC. Based on the relationship between TCF and SWRC, Lu and Dong^31^ proposed the water-retention-based unified conceptual model of TCF, which is written by

4 $$\frac{\lambda -{\lambda }_{dry}}{{\lambda }_{sat}-{\lambda }_{dry}}=1-{\left[1+{\left(\frac{S}{{S}_{f}}\right)}^{m}\right]}^{1/m -1}$$

where $\lambda$ is thermal conductivity (W/m·K) at the degree of saturation $S$, ${\lambda }_{dry}$ and ${\lambda }_{sat}$ are thermal conductivity at the dry and saturated conditions, respectively, ${S}_{f}$ and $m$ are fitting parameters related to the SWRC. ${S}_{f}$ is the degree of saturation at which the soil water retention regime is transitioned from the funicular to the pendular regime, and $m$ is the pore fluid network connectivity parameter.

## Methods

### Materials

One hundred and thirty-one nests of black and yellow mud daubers were collected from three locations in the south Louisiana (See supplementary Fig. S1 for the collection locations). Ninety-four nests were collected at Location A near the Jean Lafitte National Park, Jefferson Parish. Thirty-one nests were collected at Location B on the campus of Louisiana State University at Baton Rouge. Six nests were collected at Location C, located near the city of Lafayette. The nests were first subjected to penetrometer tests, and the resulting broken nest pieces were used to measure moisture contents, dry densities, void ratios, and specific gravities of the nests, which were reported in the previous paper^6^. The nest pieces were then dried in an oven (105 °C) for 24 h and smashed into soil particles using a mortar and pestle. Hereafter, these nest soils from Location A, B, and C will be referred to as Nest soil A, B, and C, respectively. It is worth noting that the previous study^6^ by the research team showed the dry densities and penetration resistances of mud dauber nests were comparable to the Proctor compacted reconstitute nest soil samples. This demonstrated that mud daubers do not use salivary secretions to cement soil particles and strengthen their nests. This finding supports that the nest soils used in this study can represent the mechanical and thermal properties of mud dauber nests. The specific gravity of nest soils was measured as 2.6. The grain size distributions of Nest soil A, B, and C were measured using an automated hydrometer (PARIO, Meter Group) and sieve analysis (see supplementary Fig. S2). Nest soil A, B, and C were subjected to drying cake tests, while only Nest soil A was used for direct shear, unconfined compression, and thermal conductivity tests due to the limited availability of Nest soil B and C.

The DC test results of three types of silts in Dong et al.^32^, including Bonny silts, Iowa silts, and Zhengzhou silts, were used to compare to the DC test results of nest soils, since these silts have the same soil classification as nest soils (i.e., low plasticity silts, ML). Hereafter, these three types of silts will be referred to as reference silts. The soil classifications, porosities, Atterberg limits, and soil compositions of nest soils and reference silts are presented in supplementary Table S1.

### Drying cake tests

Suction stress characteristic curve (SSCC), soil water retention curve (SWRC), soil shrinkage curve (SSC), and elastic modulus of nest soils under drying were measured using the drying cake (DC) tests^14^. The test setup was assembled following Dong and Lu^27^. The schematic view of the DC test setup is shown in supplementary Fig. S3. The DC test setup is composed of a loading system for elastic modulus measurements, an image acquisition system for measuring radial deformations of the specimen, and a transparent acrylic chamber for drying specimens at a controlled rate. Two duplicate soil specimens were used in the DC test, where one specimen was used for measuring elastic moduli and the other specimen was used for measuring its radial deformations and specimen weight changes. Two duplicate soil specimens were positioned in the transparent acrylic chamber to allow simultaneous drying at a controlled rate during the DC tests. More detailed information regarding the DC test setup can be found in Dong and Lu^27^.

Two duplicate disc-shaped specimens were prepared in two cylindrical rings with dimensions of 63.5 mm in diameter and 14.5 mm in thickness. Oven-dried nest soil (80 g) was compacted in three equal thickness layers using a metal tamper (1 kg) to achieve the targeted void ratio of 0.6, which is the average void ratio of the intact mud dauber nests^6^. It was assumed from Fig. 1a,b that mud dauber nests are fully saturated at the beginning of atmospheric drying. Thus, specimens were prepared to target the degree of saturation of 1. The compacted specimens with porous stones at the top and bottom were submerged in vacuum chambers filled with deionized and deaired water for saturation. A vacuum pressure of − 70 kPa was applied for 6 h, after which the specimens remained submerged for another 24 h to reach saturation. This procedure provides the specimens with a degree of saturation ranging from 0.94 to 1. The specimens were then extruded out of the rings and transferred onto the metal plates of the specimen holders in the DC test setup. A thin Vaseline petroleum jelly was spread on the surface of the metal plates to minimize the friction between soil specimens and plates so that soil specimens could freely deform under drying^27^. Based on this procedure, the initial porosities of the prepared specimens ranged between 0.35 and 0.39 (see supplementary Table S1).

DC tests were then started and conducted at room temperature of 20°C (± 2 °C) and relative humidity of 55% (± 10%). Measuring elastic modulus in the loading system, taking images of the specimen surface, and recording specimen weights in the image acquisition system were concurrently conducted every four hours during the tests. The tests continued until the specimen weight was constant (i.e., in equilibrium with the room humidity and temperature). The degrees of saturation at the end of the tests (reached within 72 h) were between 0.16 and 0.18 for Nest soil A, B, and C.

Young’s moduli of the specimens were measured by performing uniaxial unconfined compression tests in the loading system. The time-sequence images of the specimen surface were post-processed using the particle image velocimetry (PIV) technique to acquire radial displacement fields of the specimen. The GeoPIV-RG module^33^ was used for the PIV analysis. For the images with the pixel size of 4928 × 3264, the patch size 50 × 50 was selected in the PIV analysis. The radial displacement fields from the PIV analysis and the elastic moduli from the uniaxial unconfined compression tests were used to determine SSCC.

Lu and Kaya^14^ proposed an analytical solution based on the linear elasticity theory^34^ to determine SSCC. The analytical solution is written by

5 $${\sigma }^{s}\left(S\right)=-\frac{E(S)\cdot {u}_{r}(r,S)}{(1-\nu )\cdot r(S)}$$

where ${\sigma }^{s}\left(S\right)$ is suction stress, $E(S)$ is Young’s modulus, ${u}_{r}(r,S)$ is the radial displacement at the discretized patch with a distance of $r$ to the center of the radial displacement field at the degree of saturation $S$, and $\nu$ is Poisson’s ratio. Because suction stress is non-linearly dependent on *S*, an incremental form of the analytical solution was used to determine SSCC, which is given by

6 $$\Delta {\sigma }^{s}\left(S\right)=-\frac{{u}_{r}\left(r, S\right)}{\left(1-2\nu \right)\cdot r\left(S\right)}\Delta E\left(S\right)-\frac{E\left(S\right)}{\left(1-2\nu \right)\cdot r\left(S\right)}\Delta {u}_{r}\left(r,S\right)+\frac{E\left(S\right){u}_{r}\left(r,S\right)}{\left(1-2\nu \right)\cdot {r}^{2}\left(S\right)}\Delta r$$

More detailed information on the measurement and calculation of suction stress and validity of the DC method can be found in Lu and Kaya^14^ and Dong and Lu^27^. In the determination of SSCC, $\nu$ was assumed to be constant as 0.25 for every measured *S*^19^. However, it is important to note that $\nu$ of nest soils may be dependent on *S*^35^, which will be investigated in future study. The measured SSCCs were used to calculate the SWRCs based on the intrinsic relationship between SWRC and SSCC (Eq. 2). The calculated SWRCs were then fitted by the van Genuchten model (Eq. 3).

### Direct shear tests

The GeoJac Automated Direct Shear System (GeoTac) was used for the direct shear tests. Direct shear tests were conducted using the Nest soil A under saturated condition and 2% moisture content (average moisture content of the mud dauber nests^6^). The dimensions of the specimens were 63.5 mm in diameter and 29.5 mm in height. Oven-dried nest soil was compacted in three equal thickness layers using a metal tamper (1.3 kg) to achieve the targeted void ratio of 0.6 (average void ratio of the nests^6^). For the saturated condition, the specimens were submerged in deionized and deaired water with a level up to the specimen surface during the consolidation and shearing stages to achieve full saturation. The specimens with 2% moisture content were produced by premixing nest soil with 2% deionized water. The specimens were then stored in a sealed bag for 24 h to homogenize moisture content throughout the specimens. The saturated and 2% moisture content specimens were consolidated until primary consolidation was completed^36^, which was reached within 24 h. After consolidation, specimens were subjected to shear tests at a displacement rate of 0.013 mm/min to achieve a consolidated drained test condition^37^. In addition, the direct shear tests under different normal stresses (15, 25, 35, 50, and 70 kPa) were performed to measure the Mohr–Coulomb failure envelopes for two specimen types (i.e., saturated and 2% moisture content). It is worth mentioning that the mud dauber nests are not subjected to any external stresses (except gravity force) since mud dauber nests are in sheltered areas (e.g., under eaves and in attics) and there is no surrounding soil that can provide confining pressures.

### Unconfined compression tests

Unconfined compression tests were performed using Nest soil A to investigate the effect of moisture contents on the unconfined compressive strengths (UCS) of nest soil. The tests were also aimed to estimate the maximum UCS of mud dauber nests as the targeted dry densities of the prepared specimens for unconfined compression tests were the same as the maximum dry density of mud dauber nests (2000 kg/m^3^)^6^. The GeoJac digital load actuator (GeoTac) was used in the tests. Cylindrical specimens with the dimensions of 33 mm in diameter and 71 mm in height were prepared using the Harvard miniature compaction equipment^38^. Nest soil A was mixed with deionized water at the optimum moisture content of 12% (predetermined in the Harvard miniature compaction tests). The nest soil–water mixture was then compacted in the Harvard miniature compaction mold in four equal thickness layers. Thirty blows were applied with a 9 kg spring dynamic compaction hammer for each layer to achieve the dry density of 2000 kg/m^3^ (± 100 kg/m^3^). Specimens were then extruded and dried in an oven at 70 °C for different time durations (0.5, 1, 2, 4, 6, and 24 h) to achieve different moisture contents. After drying, specimens were cooled down at room temperature and stored for 24 h in a sealed bag to homogenize moisture content throughout the specimens. Unconfined compression tests were performed at a displacement rate of 1 mm/min. The tests were repeated at least three times for each type of specimen to confirm the validity of the results.

### Thermal conductivity function measurement

The thermal conductivity function (TCF) of Nest soil A under drying was measured using a thermal needle probe (KS-3, Meter Group). The thermal needle probe consists of a heating wire housed in a stainless-steel needle with a length of 60 mm and a diameter of 1 mm. A controller (TEMPOS, Meter Group) is connected to the thermal needle probe for thermal conductivity measurements and reading storage. Oven-dried Nest soil A was mixed with 16% deionized water (i.e., moisture content) and homogenized for 24 h in a sealed bag. The nest soil–water mixture was then compacted in the standard Proctor compaction mold in three equal thickness layers to target the void ratio of 0.6 (average void ratio of the mud dauber nests^6^). Thirty blows were applied with 2.5 kg rammer for each layer. The specimen was then extruded using an extruder. The thermal needle probe was inserted at the top center of the extruded specimen. The specimen was placed on a balance in a temperature-controlled chamber at 30°C. The balance automatically recorded the specimen weights at the same time as thermal conductivity measurements, which was used to calculate the degrees of saturation during drying. Thermal conductivities were measured every 2 h until the specimen weight reached constant (within two weeks). The TCF was then produced by plotting measured thermal conductivities versus the degrees of saturation. The measured TCF was then fitted by the water-retention-based unified conceptual model (Eq. 4).

## Results

### SSC, Young’s modulus, SSCC, and SWRC of Nest Soils

**Figure 2:**
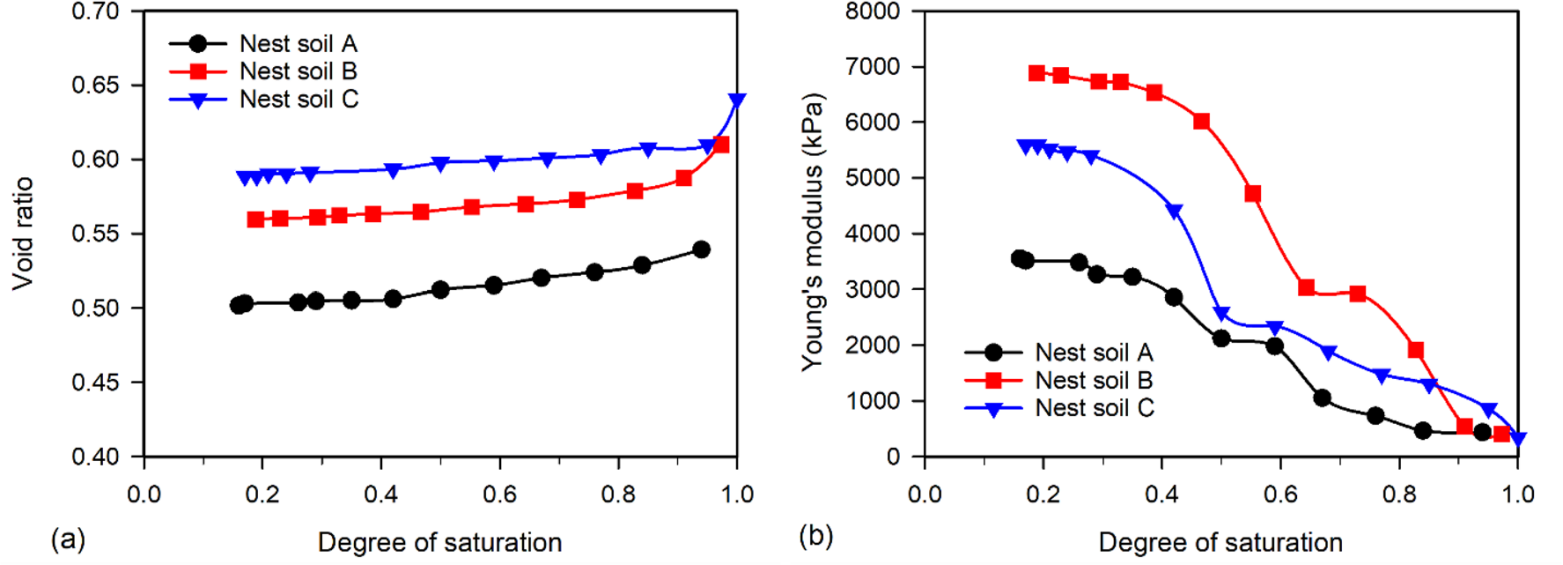
(**a**) SSCs and (**b**) variations of Young’s moduli of Nest soil A, B, and C.

Figure 2a shows the soil shrinkage curves (SSC) of Nest soil A, B, and C presented using void ratio versus degree of saturation. Void ratios slightly decreased in all nest soil specimens during the DC tests, as the specimen volume only reduced by 2.4%, 3.1%, and 3.2% for Nest soil A, B, and C, respectively. This indicates that mud dauber nests experience limited shrinkage during drying, which can reduce the potential of crack formation in the nests and control the size of nest cells for larva growth. The variation of Young’s moduli of nest soils is shown in Fig. 2b. During the DC tests, Young’s moduli increased from hundreds of kPa up to over 3500 kPa for Nest soil A, 6800 kPa for Nest soil B, and 5600 kPa for Nest soil C. The significant increases in Young’s moduli indicate that the stiffness of mud dauber nests can be significantly improved during atmospheric drying.

**Figure 3:**
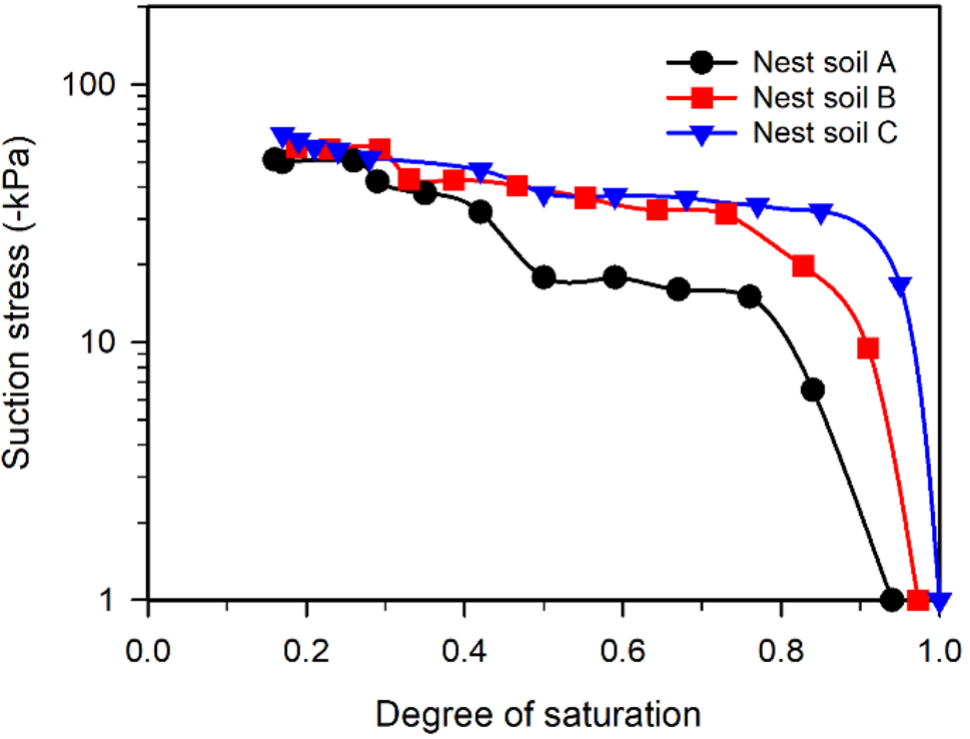
SSCCs of Nest soil A, B, and C.

SSCCs of the nest soils were calculated using the measured SSC (Fig. 2a), Young’s moduli (Fig. 2b), and the incremental form of the analytical solution (Eq. 6). The SSCCs of nest soils (Fig. 3) show that suction stresses decreased with the reduction of degree of saturation. The reduction of suction stress increased the magnitudes of effective stress in the nest soils during drying according to Eq. 1, which increased Young’s modulus (Fig. 2b) and shear strength (discussed later in direct shear and unconfined compression test results). The corresponding moisture contents at the end of DC tests ranged from 1.5 to 3% for different nest soils, which is close to the average moisture content (2%) of mud dauber nests^6^ in equilibrium with the atmospheric conditions in the south of Louisiana. This means suction stresses (ranging from -51 to -64 kPa) at the end of DC tests are approximately equal to the suction stresses in the mud dauber nests at Locations A, B, and C. The above DC test results potentially reflect the improvement of the mechanical properties of the nests under atmospheric drying.

**Figure 4:**
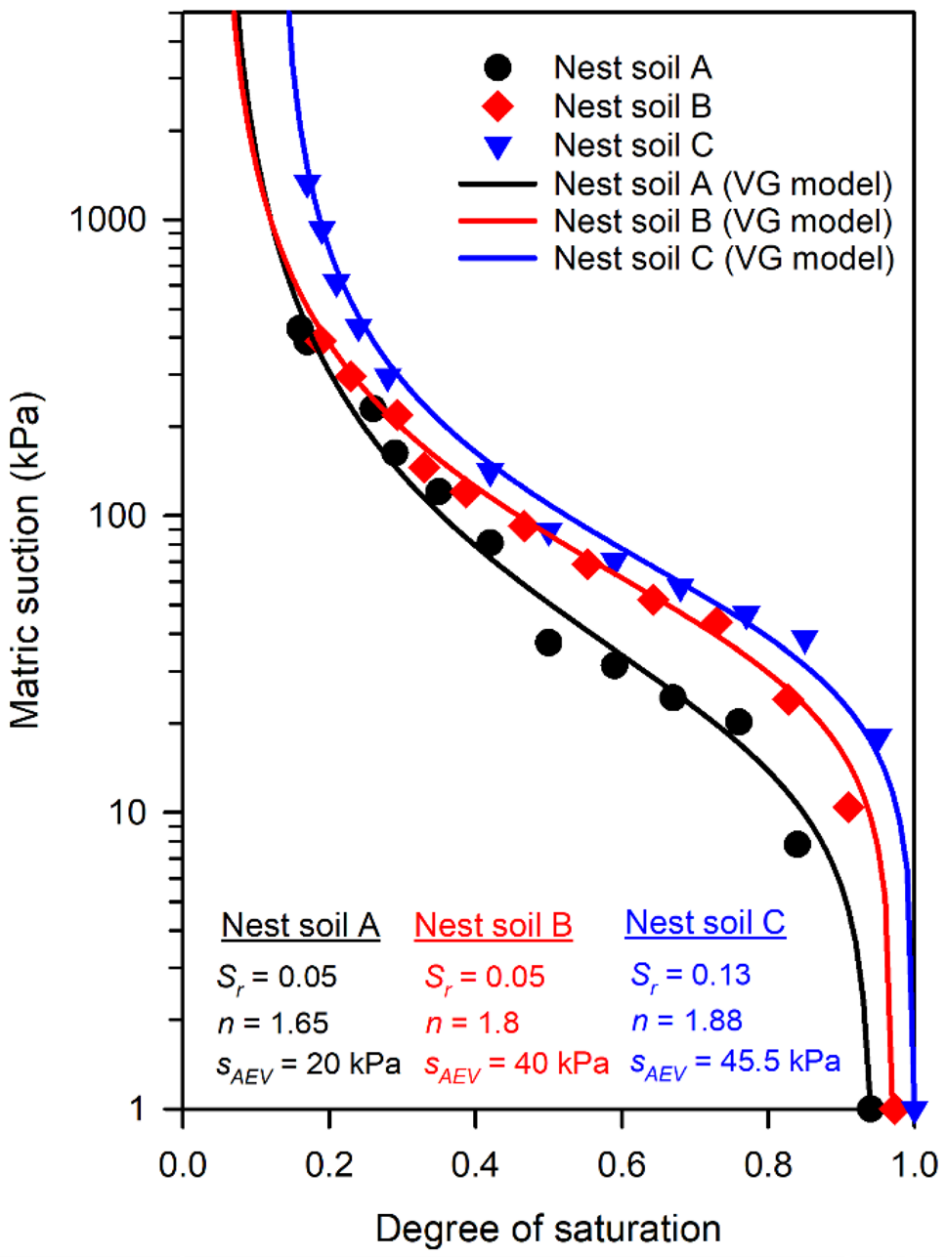
SWRCs of Nest soil A, B, and C.

The SWRCs of Nest soil A, B, and C were calculated by using the relationship between matric suction and suction stress (Eq. 2). The SWRCs were then fitted using the van Genuchten model (Eq. 3). The SWRCs and the fitted parameters *S*_*AEV*_, *n*, and *S*_*r*_ are shown in Fig. 4. The SWRCs are similar between nest soils. The Nest soil C has the highest matric suctions in the whole range of *S*. This is due to the higher portion of silt and clay particles in the Nest soil C as compared to the Nest soil A and B (see supplementary Table S1 and Fig. S2), which led to stronger adsorption and capillarity in the Nest soil C. The stronger adsorption is also demonstrated by the higher *S*_*r*_ of Nest soil C than Nest soil A and B.

### Comparisons of SSCs, Young’s moduli, and SSCCs between Nest Soils and Reference Silts

The DC test results of nest soils were compared to those of the reference silts (Bonny silt, Iowa silt, and Zhengzhou silts) reported by Dong et al.^32^. The soil classifications, porosities, Atterberg limits, and soil compositions of the reference silts are shown in supplementary Table S1. The nest soils contained more sand portion and less portion of fine-grained soil than the reference silts. The porosities of nest soils were also lower than the reference silts, meaning mud dauber nests have higher densities. The nest soils have similar Atterberg limits as the reference silts, except for Zhengzhou silt which has a lower plastic limit and higher plasticity index.

**Figure 5:**
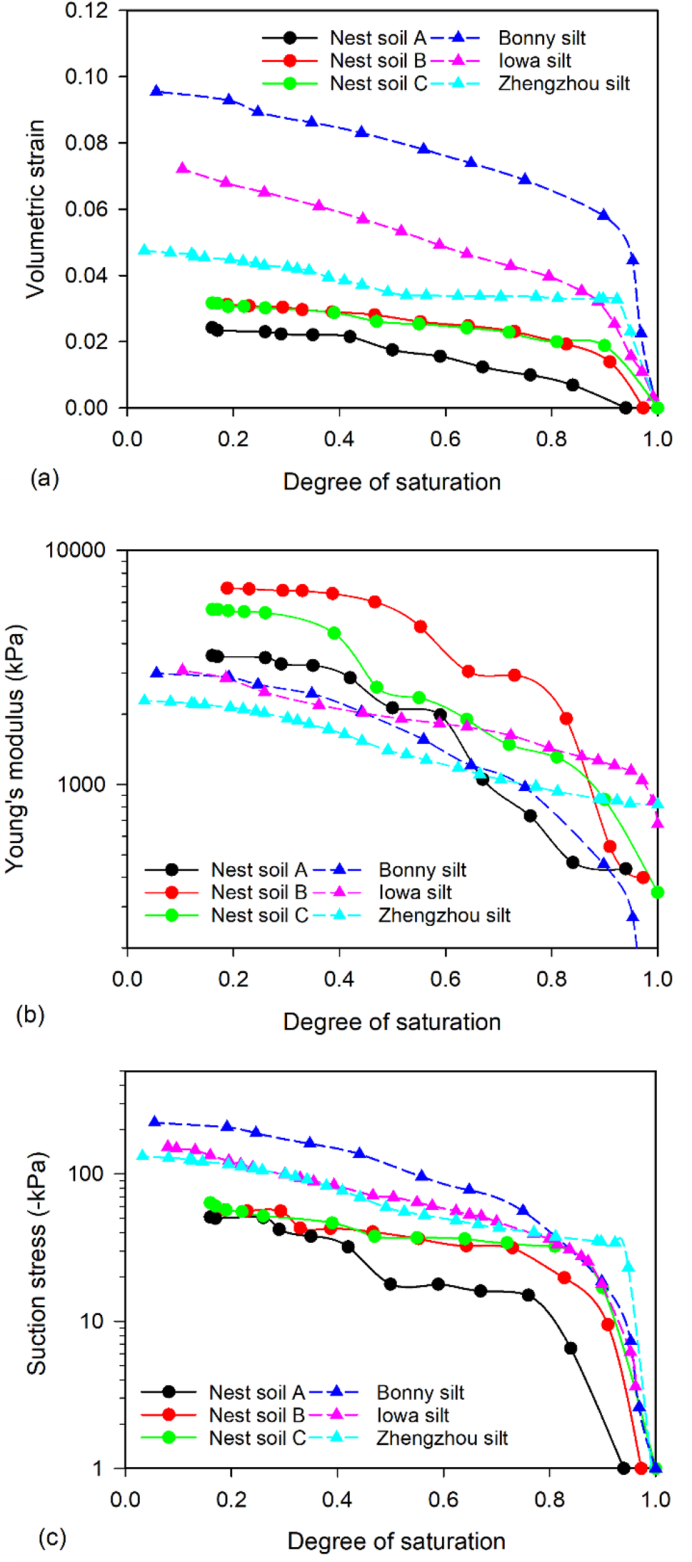
Comparisons of (**a**) volumetric strains, (**b**) Young’s moduli, and (**c**) SSCCs between the nest soils (modified from Figs. 2a, b, and 3) and reference silts from Dong et al.^32^.

The SSCs of nest soils and reference silts are presented by plotting the degree of saturation versus volumetric strain in Fig. 5a. The nest soils have similar volumetric strains between each other and lower volumetric strains than the reference silts. Young’s moduli of nest soils were also compared to the reference silts as shown in Fig. 5b. The nest soils exhibit about two to threefold higher Young’s moduli compared to the reference silts, suggesting mud dauber nests have high stiffness. The lower volumetric strains and higher Young’s moduli of mud dauber nests are attributed to the lower porosities of the nests as compared to reference silts (see supplementary Table S1). For SSCCs in Fig. 5c, the nest soils had lower magnitudes of suction stresses than the reference silts, which is probably attributed to the lower portion of fine-grained soil in the nest soils that developed lower capillary forces during drying as compared to those of reference silts. Also, according to the analytical solution (Eq. 5), suction stress is determined by the Young’s modulus and radial displacement (i.e., volumetric strain). Although Young’s moduli of nest soils were higher than the reference silts (Fig. 5b), the lower volumetric strain of the nest soils (Fig. 5a) resulted in the lower magnitudes of suction stress compared to the reference silts.

The lower volumetric strains, higher Young’s moduli, and lower suction stresses of the nest soils can be also attributed to the ability of mud daubers to select sandy silts from natural soils and repetitive tapping used by mud daubers. Park et al.^6^ compared the grain size distributions of Nest soil A, B, and C with those of natural soils at the nest soil collection site. The comparisons showed that the nest soils contained 8.5% more sand and 20% to 36% fewer clay particles than the natural soils (classified as low plasticity clay), demonstrating mud daubers can sort natural soil particles and produce sandy silts with some clay for nest construction. As compared to the reference silts, the nest soils have higher sand portions and lower fine-grained soil portions, which could result in lower suction stresses of the mud dauber nests. Also, the repetitive tapping used by mud daubers produced nests that had lower porosities (i.e., higher dry densities), which could contribute to lower volumetric strains and higher Young’s moduli of mud dauber nests as compared to the reference silts. The lower porosities and volumetric strains of mud dauber nests can reduce the potential of soil cracking during atmospheric drying and allow mud daubers to control the size of the nest cell, in which the larva can safely transform to pupa and then adult mud dauber in the nests.

### Direct shear tests

**Figure 6:**
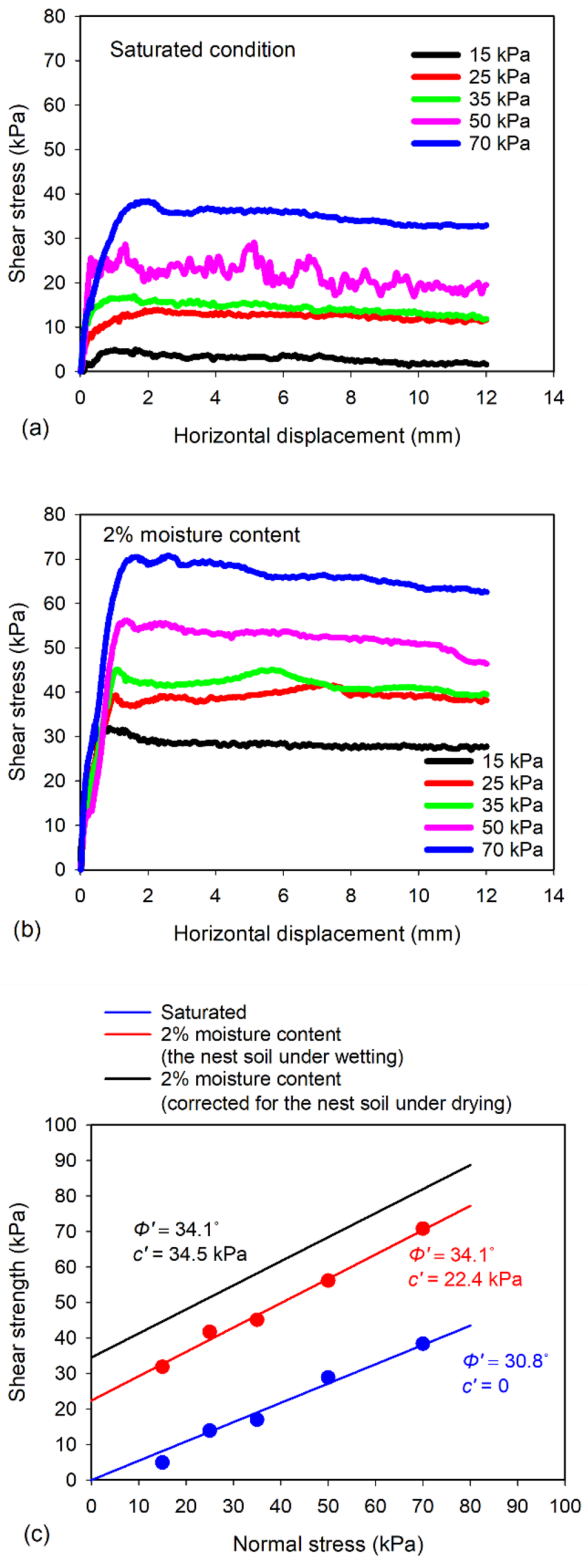
Relationships between shear stress and horizontal displacement of nest soil (**a**) at the saturated condition and (**b**) 2% moisture content; and (**c**) Mohr–Coulomb failure envelopes of nest soil at different moisture content conditions.

Figure 6a,b show the relationships between shear stress and horizontal displacement of Nest soil A under different normal stresses at the saturated and 2% moisture content conditions, respectively. Nest soil exhibits strain softening behavior under all normal stresses at both saturated and 2% moisture content conditions. At the saturated condition (Fig. 6a), maximum shear stresses were 5, 14, 17, 29, and 39 kPa at the normal stresses of 15, 25, 35, 50, and 70 kPa, respectively. At 2% moisture content (Fig. 6b), the maximum shear stresses were significantly increased to 32, 41, 45, 56, and 71 kPa at the normal stresses of 15, 25, 35, 50, and 70 kPa, respectively. The increase of shear strengths at 2% moisture content was attributed to the increase of the magnitude of suction stress with the reduction of moisture content (Fig. 3), which increased the effective stress (Eq. 1) and shear strength. Failure envelopes of nest soil at the saturated and 2% moisture content conditions are shown in Fig. 6c. Friction angle of the nest soil with 2% moisture content was 34.1°, which was 3.3° higher than the saturated nest soil. While the saturated nest soil had no cohesion, the nest soil with 2% moisture content had a cohesion of 22.4 kPa. This cohesion was generated due to the capillary forces, which is known as the apparent cohesion^15^.

However, the direct shear tests at 2% moisture content were performed using the specimen prepared under wetting condition, which does not conform with the drying condition that mud dauber nests encountered. It is well known that unsaturated soils undergoing the drying process tend to exhibit higher matric suction than the unsaturated soils undergoing the wetting process at a given moisture content due to the hysteresis effect^39^. Thus, the nest soil dried to 2% moisture content can be expected to have higher apparent cohesion than the measured apparent cohesion (22.4 kPa) of the nest soil wetted to 2% moisture content. It was attempted to predict the apparent cohesion and failure envelope of the nest soil dried to 2% moisture content by using the SSCC (Fig. 3) and the results of direct shear tests. Lu et al.^18^ proposed an extended Mohr–Coulomb failure criterion (i.e., Mohr–Coulomb failure criterion extended to the negative coordinate of normal stress in the tensile regime) to describe the shear behavior of unsaturated soils, which enables mathematical calculation of apparent cohesion as a function of suction stress (i.e., the intersection between the failure envelope and normal stress coordinate). The relationship between apparent cohesion and suction stress from the extended Mohr–Coulomb failure criterion is written by $c{\prime}={\sigma }^{s}tan\phi {\prime}$ where $c{\prime}$ is apparent cohesion and $\phi {\prime}$ is friction angle which is independent of matric suction and suction stress^40,41^. According to the SSCC (Fig. 3), the suction stress of Nest soil A was measured as -51 kPa at *S* = 0.16, corresponding to the moisture content of 3.1%. Assuming suction stresses are similar between the moisture contents of 2% (mud dauber nests) and 3.1% (DC test specimen) and friction angle is the same with the nest soil wetted to 2% moisture content^40–43^, the apparent cohesion was calculated as 34.5 kPa (i.e., -51 (kPa)·tan (34.1°)). The failure envelope of the nest soil dried to 2% moisture content can then be estimated as shown in Fig. 6c, which can be used to represent the Mohr–Coulomb failure envelope of the mud dauber nests.

### Unconfined compressive strength

**Figure 7:**
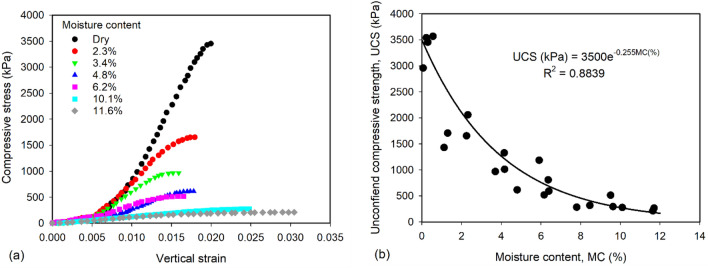
(**a**) Stress–strain relationships under unconfined compression and (**b**) UCSs of nest soil at varying moisture contents.

The stress–strain relationships of Nest soil A under unconfined compression at different moisture contents (MC) are shown in Fig. 7a. The specimens with MC ≤ 6.2% failed at vertical strain ranged between 0.015 and 0.02, while the specimens with MC = 10.1 and 11.6% failed around the vertical strain of 0.025 and 0.03, respectively. This indicates the nest soil specimens with MC ≤ 6.2% are more brittle than the specimens with MC = 10.1 and 11.6%. UCS increased from 210 to 3452 kPa as the MC decreased from 11.6% to zero (dry condition). This is because the increased magnitude of suction stress (Fig. 3) attracted soil particles together, increasing effective stress and contributing to the increase of UCS. Figure 7b shows the scattered data of UCS from the repeated tests. The UCS exponentially increased with decreasing MC, which confirms that the strength of mud dauber nests is significantly improved under atmospheric drying. It is worth recalling that the dry densities of the soil specimens used in the unconfined compression tests were around 2000 kg/m^3^, which is the maximum dry density of the mud dauber nests^6^. Thus, the measured UCSs are expected to be the maximum UCS that mud dauber nests can achieve at different moisture contents. Using the fitting equation proposed in Fig. 7b, the maximum UCS of mud dauber nests at 2% moisture content (average moisture content of mud dauber nests) was calculated as 2100 kPa.

### Thermal conductivity function

**Figure 8:**
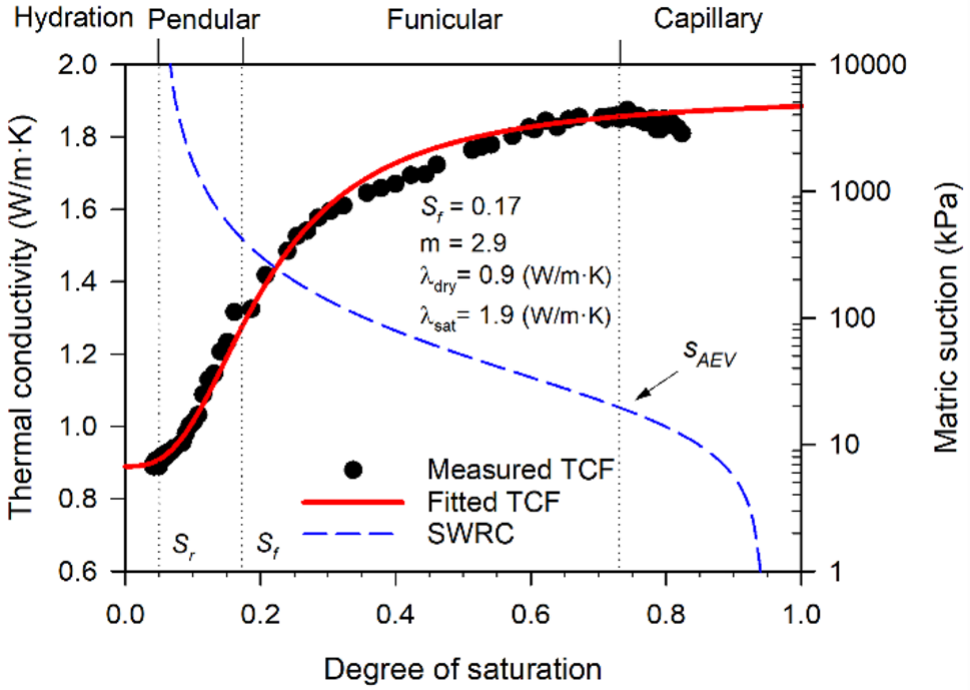
The measured and fitted TCFs and SWRC (modified from Fig. 4).

The thermal conductivity function (TCF) is presented in Fig. 8, along with the fitted curve and parameters of the water-retention-based unified conceptual model (Eq. 4). The SWRC of Nest soil A (Fig. 4) is also shown in Fig. 8 to describe the variation of thermal conductivity in relation to the soil water retention behavior. The fitted TCF covers four soil water retention regimes (i.e., capillary, funicular, pendular, and hydration). The boundaries of water retention regimes were obtained based on the van Genuchten SWRC parameters (Eq. 3) and TCF fitting parameters (Eq. 4) as shown in Fig. 8. In the capillary regime (*S* from 1 to 0.73), thermal conductivity slightly decreased. This is because air voids were trapped in water and did not affect the heat conduction at particle contacts. In the funicular regime (*S* from 0.73 to 0.17), the thermal conductivity gradually decreased until *S* = 0.4 and then substantially reduced. In the range of *S* from 0.73 to 0.4, pore water volume was reduced, and the air voids were enlarged during drying. The enlarged air voids gradually obstructed the heat conduction at particle contacts, leading to the gradual decrease of thermal conductivity. In the range of *S* from 0.4 to 0.17, the air voids continued to enlarge at particle contacts, which caused the pore water to form thin water bridges between particles. The thin water bridges provided a limited heat transfer path of solid-water–solid, which was reflected by the drastic decrease in thermal conductivity. At *S* = 0.17 (the beginning of the pendular regime, *S*_*f*_), the decreasing rate of thermal conductivity was maximum along the TCF curve. This is because the water bridges gradually disappeared in the nest soil specimen, diminishing the heat transfer paths of solid-water–solid at particle contacts. The heat transfer paths of solid-water–solid were superseded by solid-air–solid at the particle contacts, which caused the significant decrease in thermal conductivity due to the lower thermal conductivity of air compared to water (e.g., *k*_*water*_ = 0.56 W/m·K at 0 °C > *k*_*air*_ = 0.026 W/m·K^44^). In the hydration regime (*S* lower than 0.05), the thermal conductivity was almost constant. This is because the water bridges completely disappeared throughout the soil specimen, where the heat must be transferred through air voids at particle contacts. Pore water no longer affects the heat conduction at particle contacts in the hydration regime, leading to constant thermal conductivity. The thermal conductivity of nest soil at dry state was measured as ${\lambda }_{dry}$ = 0.9 W/·K, which is higher than those of the nine silts reported in Lu and Dong^31^ (ranged between 0.2 and 0.4 W/m·K). The higher thermal conductivity of nest soil at dry state is attributed to the lower porosities of mud dauber nests (ranged between 0.35 and 0.39) than those of the silts reported in Lu and Dong^31^ (ranged between 0.43 and 0.52). This is expected to cause a higher number of particle contacts and less pore space, providing more interconnected heat transfer paths.

It is also worth noting that the thermal conductivity of mud dauber nests is important to protect mud dauber larvae from the extreme temperature^3,4^. It is hypothesized that the nest soil, when collected, is near the saturated state. Thus, the thermal conductivity of the nest soil when collected can be assumed as the thermal conductivity at the saturated condition (${\lambda }_{sat}$ = 1.9 W/m·K) (Fig. 8). The thermal conductivity of mud dauber nests after atmospheric drying (average moisture content of 2%) was found to be 1 W/m·K according to the TCF. This infers that the thermal conductivities of mud dauber nests decreased by about 47% due to the atmospheric drying, which can help protect the growth of larva and pupa in the nests from the extreme atmospheric temperature.

### Bio-inspiration of mud dauber nests on sustainable earthen building construction

The nest construction of mud daubers has the potential to inspire sustainable development in the construction of earthen buildings. It is estimated that more than a quarter of the world's population is living in earthen dwellings^45^. Earthen buildings offer significant environmental advantages compared to masonry, steel, and concrete structures^46^. The raw material (i.e., soil) of earthen buildings is locally available, nonpolluting, recyclable, and has small embodied energy^47^. However, earthen construction is a labor intensive and slow construction process (e.g., significant amount of earthworks and compaction) and commonly uses Portland cement or lime to improve soil strength^48^. These practices do not satisfy the modern standards of construction productivity, consume energy intensive materials, produce high carbon footprints, and reduce the potential for recyclability, which clashes with the green credentials of earthen buildings^48^. As discussed below, the construction techniques utilized by mud daubers could potentially address those disadvantages of earthen building construction.

Mud daubers compact nest cell walls using their mandibles and front legs to produce repetitive tapping along the direction perpendicular to the cell wall (Fig. 1b). This unique repetitive tapping is intense enough to shake one’s hand when holding mud dauber with forceps^7^. The resulting dry densities of nests were similar to the Proctor compacted reconstitute nest soil samples. It is important to note that a 24.5 N rammer was used and dropped from a height of 205 mm for 75 times in the standard Proctor compaction tests, while a female mud dauber (weight less than 1 g) solitarily completed the nest construction within several hours to two days^49–52^. This possibly indicates that mud daubers use a more efficient compaction method with less energy consumption than Proctor compaction.

It is worth noting that mud daubers can select and collect sandy silt with some clay for nest construction from natural soils that were classified as low plasticity clay^6^. Although the clay portion was only about 10 to 30% in the nest soils (see supplementary Fig. S2), clay serves as the cementing agent to bond silt and sand particles and fill their pore space, which was confirmed by the SEM imaging in the previous study^6^. Clay particles in nest soils form the adsorbed water film on the clay mineral surface at low moisture content (e.g., 2%), decreasing suction stress and leading to the high strength of the nests.

Besides clay serving as the cementing agent, mud daubers also utilize atmospheric drying to improve nest strength and modulus as shown by the results of this study (Figs. 2b, 6c, and 7b). After mud daubers compact nest cell walls, the nest walls are transitioned from the plastic state to the solid state due to drying under the ambient humidity and temperature conditions (Fig. 1c). At the particle scale, fine particles (e.g., silt and clays) migrate into large pores^53^, and water menisci are being discontinuous under atmospheric drying. This leads to the increase of capillary cohesion to 34.5 kPa (Fig. 6c) and the decrease of suction stress to the range between -51 to -64 kPa at the moisture content of ~ 2% (Fig. 3). At the macro scale, the unconfined compressive strengths of the nests increased exponentially up to 2100 kPa (Fig. 7).

Since mud daubers use clay as the cementing agent and atmospheric drying to improve soil strength, it can be inferred that mud daubers build soil nests in a more sustainable manner than earthen building construction that uses Portland cement as the cementing agent. Indeed, Van Damme and Houben^48^ performed an environmental assessment and concluded that the use of Portland cement resulted in a significant increase in the embodied carbon and Global Warming Potential (GWP) of earthen materials (between 0.064 and 0.106 kg-eqCO_2_/kg). It can be seen that the maximum GWP (0.106 kg-eqCO_2_/kg) is close to the GWP of ordinary concrete (~ 0.130 kg-eqCO_2_/kg). Also, the measured unconfined compressive strengths (Fig. 7b) of mud dauber nests at moisture contents lower than 3.4% achieved similar strength ranges of earthen materials for earthen building construction^54^. Thus, it is expected that earthen materials without using Portland cement could satisfy the strength requirements of earthen buildings but also reduce the carbon footprints and increase the potential for recyclability. Future studies will focus on translating the reported nest construction techniques of mud daubers to the development of sustainable earthen building construction.

### Supplementary Information

Supplementary Information.

## Data Availability

The datasets generated during and/or analyzed during the current study are available from the corresponding author upon reasonable request.
