# Supplementary material for: Mechanical and thermal properties of mud dauber nests under atmospheric drying
Source: Sci Rep. 2023 Aug 3;13:12627. doi: 10.1038/s41598-023-39796-x (PMC10400589; doi:10.1038/s41598-023-39796-x)
Supplement: Supplementary file 1 — Supplementary Information. [file 41598_2023_39796_MOESM1_ESM.docx]

**Mechanical and Thermal Properties of Mud Dauber Nests under Atmospheric Drying**

Joon S. Park^1,+^, Hai Lin^2,*^, Hussein Alqrinawi^1,+^

Graduate Student, Department of Civil and Environmental Engineering, Louisiana State University, Baton Rouge, LA 70803, USA.

^2^Assistant Professor, Department of Civil and Environmental Engineering, Louisiana State University, Baton Rouge, LA 70803, USA.

^*^Corresponding author: [hailin1@lsu.edu](mailto:hailin1@lsu.edu).

^+^Coauthors: [jpar168@lsu.edu](mailto:jpar168@lsu.edu), [halqri1@lsu.edu](mailto:halqri1@lsu.edu).

**SUPPLEMENTARY INFORMATION**

**Supplementary Figures**

**
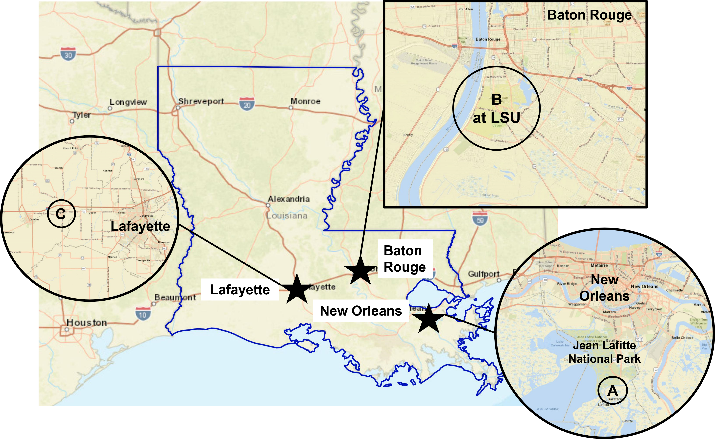
**

**Figure S1.** Sample collection map (created using ArcGIS Pro and Microsoft PowerPoint).


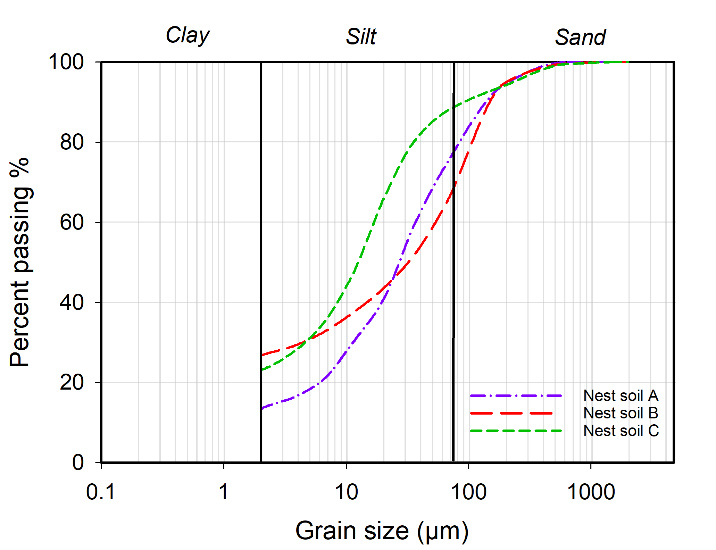


**Figure S2.** Grain size distributions of Nest soil A, B, and C.


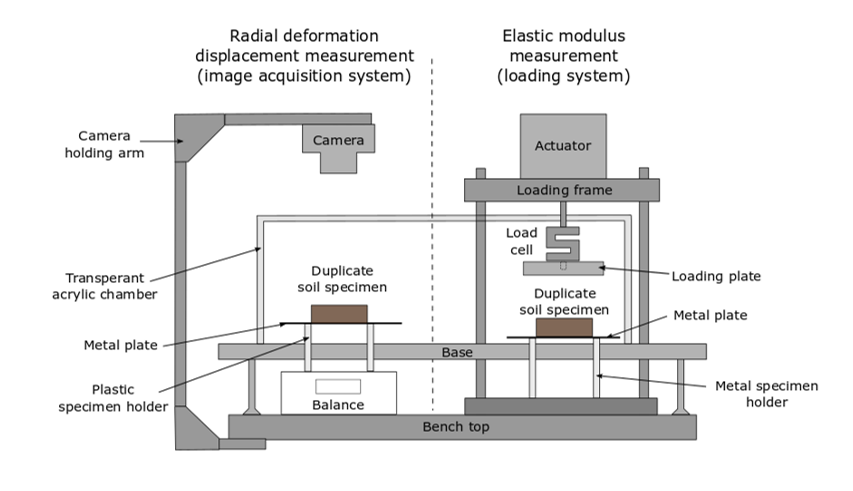


**Figure S3.** Schematic view of DC test setup.

**Supplementary Tables**

| **Table S1.** Geotechnical properties of mud dauber nest soils compared to the reference silts reported by Dong et al.^37^ | | | | | | | | | | | |
| --- | --- | --- | --- | --- | --- | --- | --- | --- | --- | --- | --- |
|  |  |  |  | Atterberg limits | | |  | Soil composition | | |  |
| Soil | Name | USCS | Porosity^a^, *n* | LL (%) | PL (%) | PI (%) |  | Sand (%) | Silt (%) | Clay (%) |  |
| Mud dauber nest soils | Nest soil A | ML | 0.35 | 24.4 | 20 | 4.4 |  | 23 | 64 | 13 |  |
|  | Nest soil B | ML | 0.38 | 25.2 | 21.5 | 3.7 |  | 32 | 41 | 27 |  |
|  | Nest soil C | ML | 0.39 | 29.9 | 23.7 | 6.1 |  | 12 | 65 | 23 |  |
| Silts^b^ | Bonny silt | ML | 0.42 | 25 | 21 | 4 |  | 10 | 78 | 12 |  |
|  | Iowa silt | ML | 0.46 | 27.4 | 21.7 | 5.8 |  | 10 | 72 | 18 |  |
|  | Zhengzhou silt | ML | 0.45 | 27.5 | 15.5 | 12 |  | N/A | N/A | N/A |  |
| ^a^Initial porosity of the specimens used in DC tests  ^b^Data from Dong et al.^37^ | | | | | | | | | | | |
